# Towards Medicines Reuse: A Narrative Review of the Different Therapeutic Classes and Dosage Forms of Medication Waste in Different Countries

**DOI:** 10.3390/pharmacy8040230

**Published:** 2020-12-01

**Authors:** Hamza Alhamad, Nilesh Patel, Parastou Donyai

**Affiliations:** 1 Department of Pharmacy, University of Reading, Reading RG6 6AP, UK; nilesh.patel@reading.ac.uk (N.P.); p.donyai@reading.ac.uk (P.D.); 2 Department of Pharmacy, Zarqa University, 132222 Zarqa, Jordan

**Keywords:** medicines reuse, medication waste, therapeutic class, dosage form, sustainability, waste management

## Abstract

Background: Medicines reuse, the idea of re-dispensing returned medicines to others following quality control, is yet to be implemented in the UK. This practice is potentially a sustainable way of dealing with returned medicines, which are otherwise classed as medication waste and destroyed. To inch towards medicines reuse, it is important to know more about the different therapeutic classes and dosage forms that make up medication waste. For example, it is helpful to know if medicines being returned are mostly solid-dosage forms and thus have the potential to be reused or are from therapeutic classes that would make medicines reuse cost-effective. Little is known about the therapeutic classes and the dosage forms of wasted medicines. This study aimed to narratively review and report findings from the international literature on the different therapeutic classes and the dosage forms of medicines that are returned by patients to community pharmacies, hospitals, general practitioners’ clinics, or collected through waste campaigns. Studies based on surveys without physically returning medicines were also included where relevant. Methods: A comprehensive electronic search of databases, including PubMed and Google Scholar, was carried out over one month in 2017 and updated by 5 November 2020, using a combination of carefully created keywords. Results: Forty-five studies published in English between 2002 and 2020, comprising data from 26 countries were included and reviewed. Oral solid dosage forms (mostly tablets) were the commonly reported dosage form of all wasted medicines in 14 studies out of the 22 studies (64%) that described the dosage form, with percentages ranging from 40.6% to 95.6% of all wasted medicines. Although there was variability among the levels of medication waste reported in different countries, findings from the UK and Ethiopia were relatively consistent; in these, medicines for the cardiovascular system and anti-infective medicines, respectively, were the most common therapeutic classes for medication waste. Conclusion: This narrative review provides insights about the different therapeutic classes and dosage forms of medication waste either returned by patients, collected through waste campaigns, or indicated in survey responses. The findings could help policy makers understand the potential implications of treating most unused medicines as medication waste and whether therefore pursuing a medicines reuse scheme could be environmentally or financially logical. The quality and the safety of these returned medicines using criteria related to the storage conditions (such as heat and humidity), physical shape (such as being sealed, unopened, unused, and in blister packaging), and tampering are other important considerations for a medicines reuse scheme.

## 1. Introduction

Waste can be referred to as any substance or object the holder discards, intends to discard, or is required to discard [1]. The World Health Organisation (WHO) defines pharmaceutical or medication waste as “expired, unused, spilt, and contaminated pharmaceutical products, drugs, vaccines and sera” [2]. Medication waste is a growing problem in the UK and different parts of the world in terms of its negative impact on governmental expenditures, the environment, and human health [3,4,5,6,7]. Waste associated with prescribed medicines cost the National Health Service (NHS) in England an estimated £300 million a year in 2009, £110 million of which related to medicines returned to community pharmacies for disposal [6]. However, the financial cost is only one part of the medication waste burden. The negative impact on the environment is also significant with one reason for finding pharmaceuticals in the water environment [7] being the improper disposal of medication waste [8,9]. The presence of medication waste in the environment can modify the physiological function of living creatures and has been linked to the possible emergence of antibiotic-resistant bacteria such as vancomycin-resistant enterococci and beta-lactam-hydrolysing Enterobacteriaceae [10], as well as the feminising effects of endocrine deactivating compounds such as ethinyl estradiol [11]. The risk to human health is not limited to pollution and contamination of the drinking water, as there is also a risk when others in the home consume unused medicines that have been stockpiled but ought to have been dealt with safely. For example, patients might self-medicate for a new illness with medication previously prescribed for a different illness, causing harm through misdiagnosis or mistreatment [12]; there might be accidental poisoning if children use stockpiled medicines; and medicine abuse might occur where the medicines are controlled or have addictive properties [13]. 

The causes of medication waste are divided into preventable (e.g., patient stockpiles medicines, overprescribing, or repeat dispensing of unwanted medicines), non-preventable (e.g., death of a patient, or a change in the prescription meaning the previous medicines are no longer required) and non-adherence behaviours [1,6,14]. Therefore, prevention is one way to reduce medication waste. Preventing waste is in fact the top option according to the Waste Hierarchy, which is a grading framework that ranks waste management options according to what is best for the environment, with “prepare for reuse”, “recycle”, “other recovery”, and “disposal” following “prevention” in decreasing preference order [15]. Many interventions have been attempted to prevent medication waste, but these have not always been effective, as the most common causes of medication waste are actually non-preventable [14]. Medicines reuse—the idea of re-dispensing returned medicines to others following quality control—is an underexplored concept in the UK but could help reduce medication waste regardless of the cause. What is more, qualitative studies have previously analysed intentions and actions towards the reusing of medication waste, reporting a possible future for the idea [16,17,18,19]. Numerous factors influence the practicalities of such an idea, including the prior storage conditions, as well as the therapeutic classes and the dosages forms of medicines considered to be waste but which might then be reused. Knowing information about the different therapeutic classes and dosage forms of medication waste creates some understanding of which medicines might potentially be up for reuse. For example, it is helpful to know if medicines being returned are mostly solid-dosage forms (thus having the potential to be reused), or liquids, injectables, etc., and whether these medicines are over the counter (cheaper/not critical to NHS costs) or other therapeutic classes that could be more relevant in terms of environmental sustainability or cost-effectiveness.

Despite a thorough literature review on the causes of medication waste [6,14,20,21,22,23], the financial [4,6,20,24,25,26,27,28] and environmental impact of medication waste [7,10,11], medicine disposal practices [8,9,22,24,28,29,30,31,32,33,34], and management strategies of medication waste [6,14], only some studies have reported the type and therapeutic classes and dosage forms of unused or returned medication waste, and none have brought the information together in a focused review [6,23,28]. This study aimed to narratively review and report findings from the literature about the different therapeutic classes and the dosage forms of medication waste that are returned by patients to community pharmacies, hospitals, general practitioners’ clinics, or collected through waste campaigns in different countries around the world. Results from studies based on surveys (without the physical return of medicines) were also included to take account of relevant data collected via this alternative method. 

## 2. Materials and Methods

A search of electronic databases was carried out over one month in 2017 and updated in 2020 ending on 5 November 2020 to identify reports and studies published in English detailing therapeutic classes and dosage forms of medication waste. Electronic databases searched comprised PubMed/Medline, Cochrane library, Grey literature (open grey and British library), National Audit Office (NAO), and National Institute for Health and Care Excellence (NICE) evidence. The bibliographies of retrieved references were also searched.

The search activity used combinations of a list of terms that included the following: types of unused medicines OR classes of unused medicines OR dosage forms of unused medicines OR types of medicine waste OR classes of medicine waste OR dosage forms of medicine waste OR types of unused drugs OR classes of unused drugs OR dosage forms of unused drugs OR types of drug waste OR classes of drug waste OR dosage forms of drug waste.

The inclusion criteria aimed to select studies published in English that reported the therapeutic classes and dosage forms of returned medication waste, either dispensed following a prescription or purchased over the counter (OTC), or a medicine sample that had expired (or had no clear expiry date) or was never fully consumed (or not used at all). Studies describing medical waste, medical device waste, and/or clinical tissue waste were excluded.

**Figure 1:**
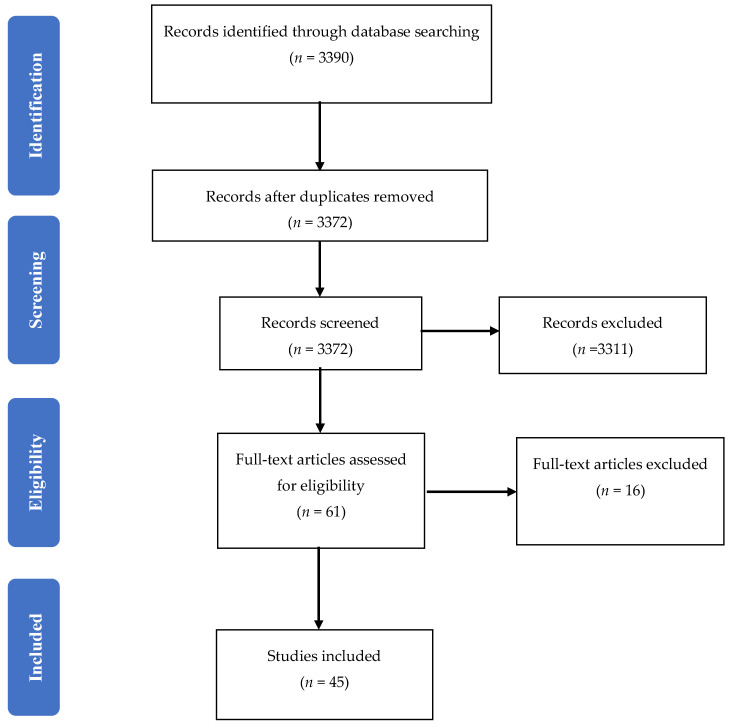
Literature search scope using the PRISMA flow chart adapted from the PRISMA Group, 2009 [35].

Study selection was completed by two researchers (H.A. and N.P.) using a Preferred Reporting Items for Systematic Reviews and Meta-Analyses (PRISMA) flow of identification, screening, eligibility, and inclusion [35] (Figure 1). At first, 3390 candidate studies were identified; then, 18 duplicates were removed. All study titles and abstracts of the remaining 3372 studies were screened, with 3311 studies removed, resulting in 61 potentially eligible studies. After a thorough full-text review of the 61 studies to assess for eligibility, 45 studies published between 2002 and 2020 were included in this narrative review. Data obtained from the retrieved studies described demographic information of the participants, the types and dosage forms of medication waste, study settings and sample size, and the time/duration of the collection of the returned medicines (varying from 4 weeks up to 12 months).

## 3. Results

The search yielded 3390 candidate studies. A total of forty-five studies published between 2002 and 2020 and comprising data from 26 different countries from around the world (Australia, Austria, Egypt, Ghana, India, Jordan, Kuwait, Malaysia, Mexico, New Zealand, Oman, Qatar, Saudi Arabia, Spain, Taiwan, Tanzania, Thailand, United Arab Emirates, United Kingdom, United States of America, China, Malta, Indonesia, Iraq, Nigeria, and Ethiopia) were included and reviewed. In some of these studies, medication waste was returned by patients to community pharmacies, general practitioners’ clinics, hospitals or sometimes collected via medicine take-back and medicine waste campaigns. However, twenty nine (the majority) studies used a survey to collect information about the therapeutic classes and dosage forms of medication waste by asking participants for information without physically collecting the waste: six studies from Ethiopia [36,37,38,39,40,41], three from India [42,43,44], two from Malaysia [24,45], two from the USA [34,46], two from Jordan [47,48], two from Egypt [26,49], two from Thailand [20,50], one from Qatar [51], one from China [52], one from Iraq [53], one from Indonesia [54], one from Nigeria [55], one from Spain [56], one from Saudi Arabia [27], one from Tanzania [57], one from Malta [58], and one from Ghana [59]. The methodologies used and the targeted populations are summarised in Appendix A Table A1.

### 3.1. Studies’ Samples

The studies’ samples were reported in different ways. Most studies reported sample size as the number of medication waste items returned or collected. Other studies reported the sample as per weight (kg), per bag, packs, or containers of the collected returned medication waste. The sample for survey-based studies was reported as the number of completed questionnaires collected or the number of participants surveyed. For more details about the sample of the studies included, please refer to Appendix A Table A1.

### 3.2. Demographics of the Participants

Gender was not reported in the majority of the studies (Appendix A Table A1). Fifteen studies (36% of the retrieved studies) described the gender of the participants, and it was not apparent that there is a gender difference associated with the presence/reporting of medication waste. For example, more women took part in seven of the studies [20,38,45,52,54,56,57] and more men took part in eight of the studies [36,37,39,40,42,49,55,59]. In the study from Egypt [49], the number of people who returned their medication waste happened to be more male than female and one study from Malaysia [24] recruited female students only. 

Age of participants was described in 23 studies out of 45 studies (51%) (Appendix A Table A1). Participants’ age profile varied in these studies and was up to 81 years. Twelve studies of the 23 studies (25%) found an apparent relationship between the mean number of returned medicinal items per patient and their age. Here, the majority of medication waste was reported to be from participants with the age ranges of 60–80 years [21,31,32,49,57]. Two studies [43,59] had more data relating to participants in the age range 20–40, but this was an artefact of the study designs, focussing on students who are likely to be in that younger age range. It is not possible to conclude that the age range of 60–80 years was associated with more medication waste as, additionally, age data was absent from half of the studies (49%).

### 3.3. Dosage Forms of Returned Medication Waste

Dosage forms were investigated in 22 out of the 45 studies (49%) on medication waste (Appendix A Table A2). Dosage forms included a range of oral solid dosage forms (tablets, capsules, granules, powders, and lozenges), liquids (syrups, injections, eye drops, suspensions, emulsions, and lotions), semisolids (ointments, creams, gel, paste and suppositories), and other items such as inhalers, sprays, patches, strips, and chewing gum. Oral dosage forms were the most commonly reported formulation in fourteen studies out of 22 (64%) with percentages ranging from 40.6% to 95.6% of all medication waste. Moreover, tablets were reported to be the most common of the oral dosage forms.

One study from Oman (60) reported that during handling of the dosage forms, most of them appeared in a suitable condition for reuse and were still in their original container. However, some had changed in colour, consistency, and odour and therefore were deemed not to be suitable for reuse. Results from a UK study [36] were consistent with the Oman study [60] in which many of the returned medication waste items were reported to be in a condition suitable for reuse as assessed by a pharmacist. These were the only two studies that directly commented on whether the medication waste returned was potentially suitable for reuse. 

### 3.4. Therapeutic Category of the Returned Medication Waste

Except for two studies [31,57] in which only prescribed medicines were included in the authors’ analysis, the majority of the studies include both prescribed and OTC medicines. Moreover, only three studies [25,26,61] included medicinal samples in addition to prescribed and OTC medicines.

The majority of the studies (42 out of the total 45) reported the therapeutic category of the medication waste, and these were included in the current analysis (Appendix A Table A2). The remaining three studies reported the medication waste individually by generic or brand name and were therefore excluded from the current analysis. 

The therapeutic categorisation systems used for reporting the findings were not the same in all studies. Seven studies used the British National Formulary (BNF) categories [6,26,49,60,62,63,64]. Seven studies used the Anatomical Therapeutic Chemical Coding (ATC) of the WHO [33,36,48,56,58,65,66]. Other ways of therapeutic categorisation included national codings such as the Saudi National Formulary (SNF) [27], Chinese Pharmacopoeia [52], and the Monthly Index of Medical Specialities online (MIMS) [20]. The remaining studies used disease and class of medicine classification such as diabetes/anti-diabetic. A breadth of therapeutic categories reported included cardiovascular system (CVS), central nervous system (CNS), alimentary tract/gastrointestinal tract (GIT), respiratory system, musculoskeletal system and joint disease, analgesics and antipyretics, non-steroidal anti-inflammatory drugs (NSAIDs), endocrine system, malignant disease and anticancer medicines, nutrition and blood, vitamins and minerals, gynaecology and medicines for urinary tract infection (UTI), antibiotics, medicines for ear, nose, and oropharynx, and skin medicines.

Eight studies out of the 42 (19%) reported that CVS medicines were the most common therapeutic category of medication waste [6,32,49,60,62,63,64,66]. Similarly, eight studies out of 42 (19%) reported that anti-infective medicines were the most common therapeutic category of medication waste [26,36,37,38,39,40,41,57]. CNS medicines were reported in five studies out of the 42 (12%) as the most common therapeutic category of medication waste [21,31,47,51,65]. 

The different therapeutic categorisation systems used in reporting medication waste (sometimes in studies completed in the same country) make the interpretation of results difficult. For example, two studies, one from India [42], and one from the USA [25], combined analgesics with nonsteroidal anti-inflammatory drugs (NSAIDs) into one therapeutic category, while five studies from India [43], the USA [34,46], Mexico [61], and Thailand [50] described analgesics and antipyretics as one category and musculoskeletal and joint disease medicines as another category. In addition, the number of studies that investigated medication waste by therapeutic categorisation was more likely to be from a small number of countries. For example, seventeen studies out of forty-two (40%) were from four countries: the UK [6,13,62,64], Ethiopia [36,37,38,39,40,41], New Zealand [21,31,65,67], and the USA [25,34,46]. This makes reporting of the results by the number of studies less representative of the international literature. 

**Figure 2:**
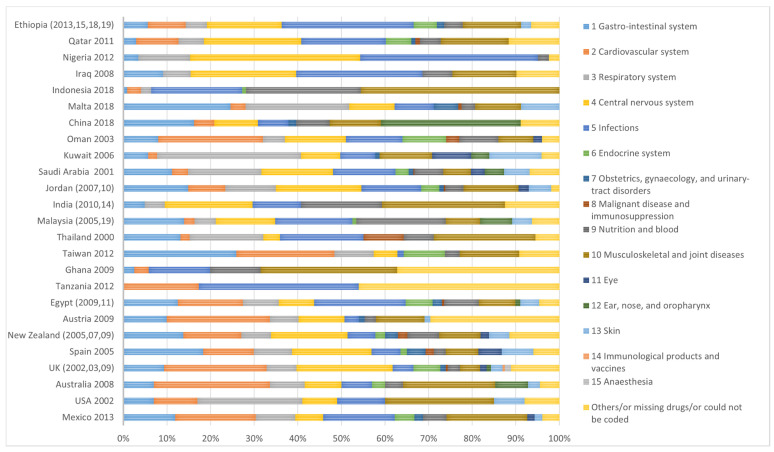
The common therapeutic categories of medicine waste reported from different countries in the world by year of data collection, re-classified according to the British National Formulary (BNF) categorisation system.

Therefore, in order to synthesise the results for this narrative review, all the different therapeutic categories were re-classified according to the BNF categorisation system and then represented by country (Figure 2). For example, NSAIDs were re-classified under musculoskeletal system medicines (BNF Chapter 10), analgesic and antipyretics were re-classified under CNS medicines (BNF Chapter 4), and alimentary tract system medicines were re-classified under gastrointestinal system medicines (BNF Chapter 1). In addition, in countries where more than one report was found, such as the Ethiopia, UK, New Zealand, Jordan, and Egypt, the sum of all returns of medication waste was calculated and reported by country.

Figure 2 shows the results of the common therapeutic categories of medication waste reported by country and after re-classification according to the BNF categorisation system. In the UK, CVS medicines were the most common therapeutic class of medication waste, with CNS medicines being the second most common therapeutic class. Other therapeutic categories of medication waste, such as gastrointestinal and respiratory medicines, were also reported but less commonly in the UK. Similar results to the UK were reported from countries such as Australia, Austria, Mexico, and Oman where CVS medicines were the most common therapeutic class of medication waste. Moreover, in Mexico, Australia, and Austria, musculoskeletal system medicines were also common and the second most reported category.

In New Zealand, CNS medicines were the most common therapeutic class of medication waste. Other therapeutic categories such as gastrointestinal, cardiovascular, and musculoskeletal system medicines (with diclofenac sodium and ibuprofen reported to be commonly returned as waste) were also reported in studies from New Zealand but less than CNS medicines. In Jordan and Qatar, results were similar to New Zealand, where CNS medicines were the most common therapeutic class of medication waste. In Jordan and Qatar, paracetamol was the most common individual tablet considered as waste. In addition, in Jordan, gastrointestinal medicines were reported as the second most common therapeutic class of medication waste followed by anti-infective medicines. In Qatar, anti-infective medicines were reported as the second most common therapeutic class of medication waste. Other therapeutic categories of medication waste such as musculoskeletal system medicines were reported in Jordan and Qatar but less commonly.

In Spain, both the gastrointestinal system and CNS medicines were the most common therapeutic classes of medication waste. In Taiwan, gastrointestinal system and CVS were the most common therapeutic classes of medication waste. While in Saudi Arabia, both the respiratory system and CNS medicines were the most common therapeutic classes of medication waste. 

In Ethiopia, Egypt, and Tanzania, anti-infective medicines were the most common therapeutic class of medication waste. The CNS medicines (in Ethiopia), and CVS medicines (in Egypt and Tanzania) were reported as the second most common therapeutic class of medication waste. Other therapeutic categories of medication waste such as musculoskeletal and gastrointestinal system medicines were reported in Ethiopia, Egypt, and Tanzania, but less so. 

Studies from the USA, Thailand, India, and Indonesia showed that musculoskeletal system medicines were the most common therapeutic class of medication waste in these countries. Finally, in Malaysia, vitamins and minerals were the most common therapeutic category of medication waste.

## 4. Discussion

Despite the extensive literature on medication waste, no literature review to date had explicitly focused on the therapeutic classes and the dosage forms of medication waste items. This narrative review addresses that gap. The principal finding is that CVS (certainly in the UK) and anti-infective (certainly in some African countries) medicines are reported as some of the most commonly returned/accumulated medication waste category. Arguably, knowing the therapeutic category of medication waste is as crucial as the quantity of the returned medication waste in terms of environmental and financial potential for medicines reuse. This is because medicines in certain therapeutic categories cost more. For example, one UK study [63] reported the volume of waste relating to respiratory system medicines to be about half (8%) that reported for CNS medicines (19%), but the cost of the medicines in the respiratory group was about the same as those in the CNS category. Thus, knowing the therapeutic categorization of medication waste makes it easier to judge where medicines reuse might be financially logical. It is of course essential to quality assure the safety of any returned medicines using criteria related to the storage conditions (such as heat and humidity), physical shape (such as being sealed, unopened, unused, and within blister packs), and tampering. Two studies conducted in Oman [60] and the UK [63] directly commented on whether the medication waste returned was potentially suitable for reuse. These studies reported that during handling of the dosage forms, most of the returned medicines appeared in a suitable condition for reuse and were still in their original container, with only a few having changed in colour, consistency, and odour; thus, these were deemed unsuitable for reuse. Findings of these studies are also important considering that unused medicines from the so-called developed world are sometimes sent for reuse to so-called developing countries.

In the UK, CVS medicines were the most common therapeutic class of medication waste. A possible explanation is that CVS medicines are one of the commonly prescribed medicines in the UK, comprising approximately 20% of all the medicines prescribed because of the prevalence of cardiovascular disease. Moreover, CVS medicines are one of the commonly amended classes of medicines because of frequent changes in doses and drugs necessitated by guidelines [62]. In Ethiopia, Egypt, and Tanzania, anti-infective medicines were the most common therapeutic class of medication waste. This is possibly because antibiotics have been available without a prescription in these countries, where also it is common for people not to complete their course of antibiotic treatment when their symptoms resolve. In New Zealand and Jordan, CNS medicines were the most common therapeutic class of medication waste with paracetamol as the most common individual tablet returned as waste. The potential explanation here is that analgesics (with paracetamol reported to be the most common) are frequently used for the self-medication of headaches, which is a commonplace discomfort. Similarly, in the USA, Thailand, and India, the musculoskeletal system medicines were the most common therapeutic class of medication waste with NSAIDs being the most common group reported in these countries, again reflecting their common usage. In the study from Malaysia, vitamins and minerals were reported as the most common therapeutic category of medication waste, but this is likely an artefact of the methods, which only sampled female students.

This narrative review synthesised information about the most commonly found medication waste products from different countries around the world. However, the results should be interpreted carefully. First, the findings apply to medication waste that was returned by patients only or reported in surveys and does not take into account the substantial amount of medication waste likely to be disposed of into household garbage or via the sink or simply kept stockpiled unreported at home [65]. Second, the small sample size and the small number of returns of medication waste in the majority of the studies made these studies less likely to be representative of a global picture. Third, the CNS classification of paracetamol as the most commonly reported item as waste raised the percentages of waste from the CNS therapeutic class compared to other therapeutic classes such as musculoskeletal, alimentary tract, and respiratory systems. Paracetamol is considered cheap, and one may argue that it is not worthwhile to set up a medicines reuse system if this is the largest category of returned medicines in any one country. Fourth, the quality of the studies included in this narrative review was not checked because of the disparity of methods and a lack of specific criteria, which could affect the results reported (as none of the papers found were excluded) and could be seen as another limitation of this study. Finally, the results of this narrative review cannot be generalised. For example, results from Ethiopia, Egypt, and Tanzania of having antibiotics as the most common therapeutic category of medication waste cannot be generalised (although reported to be the commonest along with CVS medicines) to other countries where antibiotics are only available with a prescription such as in the UK or the USA. In addition, results from the two Malaysian studies, which reported that vitamins and minerals were the most common therapeutic category of medication waste, is impossible to generalise to the larger population, as one study was only completed with female Malaysian students (no males). The other Malaysian study was also completed by more females than males. 

This narrative review has other limitations that should also be acknowledged. First, it included results from reports, theses, audits, and the grey literature, but there is always a risk that some studies were not included as a result of not performing a thorough enough systematic search. Second, the search strategy was restricted to studies that were published in the English language only and so could have missed other valuable research. Third, the reasons behind the accumulation of the returned medication waste from each therapeutic category were not clearly evidenced in all the studies. Some studies provided possible explanations that may apply only to the country from which data were obtained, and therefore, it may not be appropriate to generalise these explanations. Finally, information about what motivates people to return their medication waste and if they returned a certain type of medication waste over others were not investigated and remain unknown. 

This review is the first to provide narrative information about the different therapeutic categories and dosages forms of returned medication waste from different countries around the world. Pooling information about the different therapeutic classes and dosage forms of medication waste can help increase knowledge about medicines that are returned unused and or otherwise classed as wasted medicines, so that extrapolations can be made about the costs of waste and whether it is worthwhile to reuse these medicines. For example, paracetamol is considered cheap, and one may argue that it is not worthwhile to set up a medicines reuse system if this is the largest category of returned medicines in any one country. In addition, oral solid dosage forms are more likely to be suitable candidates for reuse compared to other dosage forms such as liquids or injectables; therefore, it is useful to know where this is the most commonly returned formulation. However, a pharmacist’s hand inspection of such medicines would not be sufficient to address concerns about the quality and the safety of returned medicines. For example, there would also need to be additional checks in place for storage conditions (e.g., under excessive heat and humidity), and physical characteristics (such as being sealed, unopened, unused, and being in a blister) which could indicate (non-)tampering, degradation or contamination, in addition to the visual indicators. These concerns would need to be addressed before medicines reuse becomes a reality [23,68], and one way to do this would be through the application of technology [69,70].

This narrative review identified a large number of studies from the literature that investigated the different therapeutic classes and the dosage forms of medication waste returned by patients to healthcare settings, and through waste campaigns, as well as information obtained from survey responses. Although there was variability among the levels of medication waste reported in different countries, findings from some countries such as the UK and Ethiopia were relatively consistent and appeared to reflect the local usage of these medicines. This suggests that medication waste categories might be proportional to the prevalence of medicines in each specific country, which remains to be tested in future studies. Future studies that focus on assessing the quality and the safety of retuned medicines, and exploring public and healthcare providers’ perception about medicines reuse should also be performed before medicines reuse becomes a reality. For example, contained sites such as long-term care facilities or hospice care settings where the medications are presumably stored correctly might be more capable of reusing medicines and could be a realistic site for trialling medicines reuse in the future. 

## 5. Conclusions

Findings from this narrative review provide insight about the different dosages forms and the therapeutic classes of medication waste, which can be used to support future medicines reuse-related research and explorations. It appears that the therapeutic categories of medication waste are reflecting prevalence of usage, inviting policy makers in each country to reflect on whether medicines reuse could be cost-effective in their own settings. Any medicines reuse scheme would still need to consider quality and safety checking of returned medicines over and above the pharmacists’ visual checks.

## Appendix A

**Table A1:** Summary of the research instrument, sample, and demographics of the included studies.

Year of Study	Author(s)	Country/Settings	Research Instrument	Wasted Medicines Information (e.g., Take Back Campaigns vs. Survey)	Sample	Demographics
2015	Gracia-Vásquez et al. [61]	Mexico; nine cities of Monterrey	Unused/expired medications were collected from households in a special container placed in a visible and accessible location in 85 collection centres in community pharmacies located in nine cities in the Monterrey metropolitan area over 12 months from March 2012 to February 2013	**Take back** program.	Random sample of 22,140 items, 30% of total drugs collected over 12 months), as 70% were unable to be classified.	Not studied.
2008	Braund et al. [21]	New Zealand	Over a five-week period medications were returned to two collection point pharmacies and questionnaires were completed by returners.	**Take back** program. In addition, a **questionnaire** was completed to determine the reasons that the medications were not used.	163 returns, comprising of 1399 items, with only 126 returned questionnaires.	The majority of those returning medications fall within the age range of 61–80 years.
2007	Braund et al. [67]	New Zealand; Otago Pharmacies	Medications returned unsolicited to Otago pharmacies over a 9-month period, from 1 April to 31 December 2005.	**Take back** program.	A random sample (159 kg, 12%) of the 1294 kg of medications returned for destruction over a nine-month period from the Otago region were identified.	Not studied.
2009	Braund et al. [31]	New Zealand; Hutt Valley District Health Board	A Disposal of Unwanted Medication Properly (DUMP) campaign was conducted for 4 weeks in November 2007 in 31 community pharmacies. Questionnaires were completed by the returners.	**Take back** program. ‘Disposal of Unwanted Medication Properly **(DUMP)’ campaign**.	Of the total 1605 bags returned over 4 weeks for disposal, only 329 bags (20%), containing a total of 1253 items were fully analysed. Only 653 questionnaires were completed (41%).	The age distribution of the patients with unused medications was <20 (8%), 21–40 (13%), 41–60 (28%), 61–80 (40%) and >81 years (11%).
2010	Caroline et al. [29]	New Zealand; Nelson Bays region	A Disposal of Unwanted Medication Properly (DUMP) campaign was conducted for 5 weeks in November and December 2009 and for 3 weeks afterwards. Surveys were completed in 379 bags.	**Take back** program. ‘Disposal of Unwanted Medication Properly **(DUMP)’ campaign**.	Of the 6500 DUMP bags distributed across the Nelson Bays region, 1244 bags were returned (response rate 19%), with an average of 7 items per bag (number of items returned 8609).	Not studied.
2009	James et al. [65]	New Zealand: Taranaki region (around 37,000 households)	Unused medications returned for disposal to the 24 community Pharmacies in the Taranaki region (≈37,000 households) of New Zealand over 6 weeks.	**Take back** program.	716 individuals returned 3777 items of unused medications. Of the 3777, information for the amount issued and returned was complete for 2704. The majority (51%) of returns contained 75–100% of the original dispensed amount of medication.	Not studied.
2005	Langley et al. [62]	United Kingdom; East Birmingham	Unused medications returned to 8 community pharmacies and 5 general practices (G.P.) surgeries over 4 weeks each (4 weeks during August 2001, 4 weeks during March 2002, respectively).	**No return campaign** was conducted and no attempt was made to encourage patients or carers into returning medicines. Medicines returned to 8 community pharmacies and 5 general practices (G.P.) surgeries over 4 weeks were assessed.	A total of 114 returns; 24 (21.1%) to G.P. surgeries and 90 (78.9%) to community pharmacies. The total returns comprised 340 items, of which 42 (12.4%) were returned to G.P.s and 298 (87.6%) to community pharmacies.	Older patients (60 years and over) returned 61.4% of items with 24.6% of returns coming from patients aged 30–59 years and 5.3% of returns originating from patients under 30. Ages were not recorded for 8.7% of returns.
2007	Mackridge et al. [63]	United Kingdom; Eastern Birmingham Primary Care Trust (P.C.T.)	Unused medications returned to pharmacies and G.P. surgeries were collected over 8 weeks in May and June 2003 in Eastern Birmingham Primary Care Trust (P.C.T.). Three-quarters of the P.C.T. sites participated, 51/60 (85%) pharmacies and 42/61 (70.5%) G.P. surgeries.	Unused medicines were **returned** and data were collected in Eastern Birmingham Primary Care Trust (PCT), a predominantly urban PCT with an ethnic minority population of 20%.	934 return events were made from 910 patients (190 GP surgeries, 744 pharmacies), comprising 3765 items (431 GP surgeries, 3334 pharmacies) and totalling 4934 individual packs.	The mean age of 63.5 ± 0.78 years (10 months to 99 years) and there was no detectable correlation between the mean number of items returned per patient and their age.
2008	Bradley [64]	United Kingdom; Cumbria	A medicine waste audit in community pharmacies of Cumbria where each pharmacy asked to analyse 20 returns of unused medicines. Further qualitative data were collected by interviewing the patients and their representatives.	Unused medicine were **returned** to community pharmacies in Cumbria where each pharmacy was asked to analyse 20 returns of unused medicines.	A total 4563 items was received from 87 community pharmacies across Cumbria.	Not studied.
2010	Trueman et al. [6]	United Kingdom	Unused medications returned to 114 pharmacies (51 from London/urban, 32 from North-West/rural and urban, 24 from Yorkshire and Humber/rural and urban, 7 from West-Midlands/rural) from 5 primary care trusts.	Unused medicine were **returned** to 114 pharmacies in 5 primary care trusts.	In total, 8626 items were reported as returned with 7500 of the returned items identified and coded for analysis.	Not studied.
2008	Coma et al. [56]	Spain; Barcelona	Unused medications returned to random sample of 118 community pharmacies in Barcelona invited to participate, 38 (32%) agreed to participate. Data were collected from February to April 2005. Questionnaires were completed by the returners.	Unused medications were **collected** from 38 community pharmacies over a period of 7 consecutive working days (excluding Sundays). A **questionnaire** was designed to evaluate each returned medicine.	In total, 1176 packages were returned by 227 patients. The majority were medicines (96.6%), and the rest were medical supplies or devices (0.5%) or other products sold in the community pharmacy (2.9%; e.g., personal care, nutrition). Most medicines returned were drugs for human use (99.8%) and only 0.2% were for veterinary use.	54.6% women, 64 ± 20 years old.
2015	Law et al. [46]	U.S.A.; Southern California	Cross-sectional, observational two phases study was conducted using a convenience sample in Southern California. In Phase I, a web-based survey was conducted at one health sciences institution; and in Phase II, a paper-based survey at drug take back events.	Web and paper-based **survey**.	Phase I: A total of 539 prescription medications were reported, with an average of 4 per household. Approximately 7% of the unused medications were expired, and 30% were brand name. Phase II: Of the 776 unused medications returned for disposal, 311 (40%) medications were brand name. Nearly two-thirds (66.2%) were expired, discontinued by the physician (25%), or became unused after the patient indicated feeling better (17.6%).	Phase I: Average household age was 36.4 years, but not described in Phase II which the drug take back program.
2004	Garey et al. [25]	U.S.A.; Houston, Texas	Unused medications returned to community pharmacy during “Medicine Cabinet Clean up Campaign” over 6 months between April and September 2002 (pilot study).	“Medicine Cabinet Clean up **Campaign**”	In total, 1315 medication containers were returned to the community pharmacy. 63% of returned medications were dispensed between 2000 and 2002, 31% from 1995 to 1999, and 6% before 1995.	Not studied.
2015	Maeng et al. [34]	U.S.A.; Regional health plan in Central Pennsylvania	Telephone survey conducted by a survey research centre.	Telephone **survey**.	Not studied.	Not studied.
2014	Vogler et al. [66]	Austria; Vienna	Unused medications collected from household garbage in all districts of Vienna between 12 October and 24 November 2009.	**Unused medicines** ending up in household garbage were **analysed** in all districts of Vienna.	In total, 152 packs were identified from manually investigated sample from household garbage in Vienna.	Not studied.
2013	Chien et al. [71]	Taiwan; Shuang-Ho university teaching hospital	Discarded drugs were collected from the Drug Discarding Bin at the Shuang-Ho Hospital over 4 weeks.	**Discarded** drugs from the Drug Discarding Bin at the Shuang-Ho Hospital in Taiwan were **collected** and **analysed**. A **paper**-based **questionnaire** was utilised to study the attitudes and perspectives of the out-patients and/or patients’ family members about discarding unused medications that were prescribed and covered by the National Health Insurance policy.	A total of 98 kg (51,972) discarded medications collected from the hospital Drug Discarding Bin.	Not studied.
2013	Abushanab et al. [48]	Jordan; Amman	Cross sectional survey using a pre-piloted questionnaire was used in the interview of 219 households in 9 areas of Amman to about the types of drugs stored at home conducted between November 2009 and April 2010.	**Survey** study.	From the 2393 drug products presented in surveyed households, 24.99% was considered as drug waste (drug wastage, calculated as the sum of drug products that had expired 10.91%, had no clear expiration date 1.84%, or which had never been used since dispensing 15.04%).	Age of the interviewee (years) 42.15 ± 14.67.
2012	Al-Azzam et al. [47]	Jordan; North of Jordan particularly Irbid	Validated questionnaire was administered to 435 households selected randomly from different areas in the north of Jordan (particularly in Irbid governorate) in the period from April 2007 and until August 2007.	**Survey** study.	Of the total of 2835 medication items found in the 435 selected houses, 65.3% were in use, and 34.7% were not in use.	Age of the interviewee (years) 36.4 (±11.9).
2002	Abou-Auda [27]	5 regions in Saudi Arabia and other Gulf countries (Kuwait, U.A.E., Qatar, and Oman)	A questionnaire was administered to a total of 1641 households participated in the study (1554 in Saudi Arabia; 87 in other countries).	**Survey** study.	A total of 12,463 drug products were found in 1554 households in Saudi Arabia. Among the 87 households surveyed in the 4 other Gulf countries, 616 drug products were found.	Not studied.
2011	Kheir et al. [51]	Qatar	This was a cross-sectional, exploratory, descriptive study. Households included in the study were identified using a list of home telephone numbers was selected randomly from the telephone directory maintained by Qtel^®^, Qatar’s national telephone company.	**Survey** study.	Four hundred and thirty-two phone calls were made to invite respondents to take part in the study. Eighty-one household representatives initially expressed interest in being part of the research during the first call, of whom 49 participants (18% of the targeted sample size) answered all survey questions.	Not reported.
2007	Al-Siyabi et al. [60]	Oman; Sultan Qaboos University Hospital (SQUH)	Observational study of returned unused medicines to the pharmacy at SQUH between February and June 2003.	**Returned** medicines received by pharmacy staff were analysed in the study.	A total of 1171 items (medications) were returned to the pharmacy at SQUH; among these, 99 drugs were excluded. Medicines were included only if they had SQUH patients’ labels, and any items without SQUH patient’ labels were excluded from study.	Not studied.
2004	Wongpoowarak et al. [20]	Thailand; Songkhla	A cross-sectional survey of unused medicines of a random sample of 931 households in the Songkhla. Of the 931 households surveyed and interviewed by using a structured questionnaire, there were 453 (48.7%) where at least one person reported having unused medications.	**Survey** study.	A total of 1004 unused medication (items) were identified from 523 respondents who had unused medications in 453 households. Nine items could not be identified because their physical appearance did not match that of any known medication. Thus, 995 items were included.	Gender: Male: 224 (42.8%). Female: 299 (57.2%). Age: 0–9 years: 167 (31.9%). 10–19 years: 52 (10.0%). 20–29 years: 66 (12.6%). 30–39 years: 76 (14.5%). 40–49 years: 64 (12.2%). 50–59 years: 40 (7.7%). ≥60 years: 58 (11.1%).
2013	Sooksriwong et al. [50]	Thailand; 4 regions of Thailand: Bangkok, Chiang Mai, Khon Kaen, Mahasarakham and Songkla	Structured questionnaire developed to survey 357 households which were interviewed and during January and March 2011: 46% in Bangkok and 54% in upcountry.	**Survey** study.	2208 drug items were found in 357 households. 952 items (43%) of these drug items were dispensed by public hospitals, 750 items (34%) were from drug stores, 163 items (8%) were from private hospitals and 210 items (10%) were from others.	Not studied.
2011	El-Hamamsy [26]	Egypt; Cairo	Pilot study where all drugs returned unused to 20 community pharmacies in Cairo over period of one month (April 2009).	All drugs **returned unused** to 20 community pharmacies located in Cairo, Egypt were documented during April 2009. A total of 316 patients completed a survey about medication disposal practices.	A total of 541 drugs were returned and collected over one month.	Not studied.
2012	Ibrahim et al. [49]	Egypt; Alexandria	A cross-sectional descriptive study where all drugs returned unused into randomly selected 60 pharmacies in Alexandria over a period of one month during March 2011.	**Survey** study.	A total of 657 drugs were returned from 600 patients to the 60 pharmacies over one month.	Males constituted the higher percentage of the participants 56.7%. Elderly having 60 years or above constituted the highest proportion of the sample (28.3%), while the lowest percentage (4.0%) was within the age group (10 to less than 20).
2010	Guirguis et al. [32]	Australia; St Vincent’s Hospital, Melbourne	Retrospective audit looked at all expired medications or those no longer needed were collected at St Vincent’s Hospital, Melbourne over 2 months (July and August 2008).	**Retrospective** audit looked at all the items **collected** over a period of 2 months: July and August 2008.	A total of 293 items were collected from 40 patients recruited over 2 months.	Older than 65 years of age.
2014	Kagashe et al. [57]	Tanzania; tertiary hospital in Dar ES-Salaam city	Cross-sectional study carried out at a tertiary hospital in Dar es Salaam city Tanzania where patient files were analysed for last admission treatment information for the year 2012.	**Survey** study.	About 56.3% of medicines prescribed were dispensed to patients. Out of the total 1418 dispensed drugs, 730 medicines were wasted.	The mean age of the study population was 44 years, with minimum age of 11 years and maximum of 88 years. Medicines wastage was reported from female more than in male (404 (55.7%) vs. 326 (47.1%), respectively).
2007	Abahussain et al. [33]	Kuwait; Kuwait city	Municipal collection program of unwanted medicines from households in Kuwait City.	**Take back** collection program.	Sample of 200 households in Kuwait received an educational letter and special plastic bags in which to place unwanted medicines to be collected by the municipality. A second convenience sample of an additional 14 households in Kuwait received the same educational letter together with a face-to-face interview and assistance in collecting unwanted medicines.	Not studied.
2013	Aditya [43]	India; dental hospital in North India	Descriptive cross-sectional survey of dental students based on a structured questionnaire format) was carried out in a teaching dental hospital in North India.	**Survey** study.	244 students, with 8 students were excluded due to incomplete forms only 236 were included.	Age of participants from 20 to 40 years.
2011	Gupta et al. [42]	India; Greater Noida City	A simple randomised prospective survey study that was carried out for a period of six months in selected areas of Greater Noida City. Randomly selected 102 houses were visited to educate and assess the people about Home Medicine Cabinet.	**Survey** study.	A total of 392 people were surveyed in 92 houses with the exception of 10 houses.	Of the total 392 people surveyed: The male vs. female for those with age >12 years is 144 (36.73%) vs.133 (33.93%), respectively. The male vs. female for those with age <12 years is 69 (17.6%) vs. 46 (11.74%), respectively.
2014	Mirza and Ganguly [44]	Anand district of Gujarat, India	A cross-sectional study was conducted during 2012–2014. Data were collected from 800 houses, 400 each from urban and rural areas and then analysed for the details of medicines available in the house.	**Survey** study.	A total of 800 houses, 400 each from urban and rural areas, were included for the study, which was based on the prevalence of self-medication as per a previous study done in India.	The participants above the age of 18 years, capable of giving information of medicine use within the family (the heads of the households or their spouses or any adult capable of delivering required information) were interviewed for the study. The presence of any healthcare professional amongst the family members in a visited house was excluded in order to avoid biased answers.
2009	Ali et al. [24]	Malaysia; Universiti Sains	A prospective descriptive, cross-sectional survey was conducted from February to June 2005 in the Universiti Sains, Malaysia.	**Survey** study.	A total of 481 single female respondents were targeted for a questionnaire-based survey on randomly sampled students. A total of 1724 different types of medicines were found with an average number of 4 medicines found per student.	Respondent were only females ages varied from 19 to 54 years old. 89.2% (*n* = 429) of the students were categorised in the 19–24 years age category, while 8.7% (*n* = 42) were aged between 25 and 30 years old. The remaining 2.1% (*n* = 10) were aged between 31 and 54 years.
2020	Hassali and Shakeel [45]	Selangor, Malaysia	The quantitative, cross-sectional study was conducted by face-to-face interviews using a pre-validated structured survey form in Selangor, Malaysia from September to December 2019.	**Survey** study.	Among the approached 600 individuals, 426 showed their willingness to participate in the study. Hence, the response rate of the present study was 71%.	A large proportion of the respondents (269; 63.1%) were females. Most of the respondents were Malay (378; 88.7%), followed by Chinese (32; 7.5%). The study population included students, private and public sector employees, and housewives, who were over 18 years of age. More than half of the respondents were bachelor’s degree holders (220; 51.6%).
2014	Aboagye et al. [59]	Ghana	The study was conducted over selected areas in Ghana with a questionnaires were randomly issued out from 13 to 20 December 2009.	**Survey** study.	Out of the 200 questionnaires sent out, 180 were retrieved and analysed.	The majority of the respondents 62.8% (113/180) were between the ages of 21 and 40 years, and the minority 5.6% (10/180) were above 61years. A total of 99 (55%) of the respondents were males corresponding to 81 (45%) females.
2019	Huang et al. [52]	China, six provinces in North, Central, and Southern regions of China	A cross-sectional survey of 625 households survey was carried out between March and April 2018 in China.	**Survey** study.	We randomly sampled 1000 households from the communities according to community population information registration forms. At the end of the period, after removal of incomplete responses, a total of 625 completed and usable questionnaires were received, equating to a response rate of 62.5% (625/1000).	The majority of respondents, 61.9% (387/625) in the households visited were females. A high proportion 60.6% (379/625) of the respondents were employees from different companies. In terms of age groups, 78.4% (490/625) of respondents were less than 30 years old, and 12.0% (75/625) of the respondents were aged between 31 and 45.
2019	Vella and West [58]	Malta, Maltese village	The study was conducted from 1 April to 31 December 2018 within a community pharmacy in a small Maltese village with 3500 inhabitants.	**Survey** study.	A total of 411 medications were collected, amounting to a total cost of approximately €2600.	Not reported.
2020	Insani et al. [54]	Bandung, Indonesia	A descriptive cross-sectional study was conducted in Bandung, Indonesia, from November 2017–January 2018.	**Survey** study.	A total of 497 respondents completed the questionnaire.	A total of 497 respondents completed the questionnaire of which many were female (*n* = 366, 73.6%) and aged between 18 and 30 years (*n* = 424, 85.3%). More than half of them completed secondary education (*n* = 326, 65.6%) and about one-third (*n* = 167, 33.6%) were university graduates. A large proportion of respondents were students/university students (*n* = 342, 69.0%).
2010	Jassim [53]	Basrah, Iraq	This is a descriptive study involving a questionnaire survey to determine the extent of drug storage and self-medication in 300 household units Basrah, Iraq between 2007 and 2008.	**Survey** study.	A total of 300 household units in Basrah, Iraq included in this study. A survey was conducted in 300 households in Basrah, southern Iraq to determine the availability, source, and storage conditions of medicinal drugs and the prevalence of self-medication with antimicrobials.	Not reported.
2012	Auta et al. [55]	Nigeria	A cross-sectional survey of a random sample of 240 undergraduate pharmacy students of the University of Jos, Jos, Nigeria, was carried out.	**Survey** study.	A total of 240 students were chosen randomly with at least 50 from each professional level/year to participate in the study. A pre-tested, self-administered questionnaire was distributed among participants after explaining the purpose of the study and obtaining oral informed consent.	A total of 188 of the 240 (representing 78.3%) questionnaires administered were completely filled and returned by respondents. The respondents consisted of 55.3% males and 44.7% females with the majority of the respondents between the ages of 21 and 25 years.
2015	Wondimu et al. (41)	Tigray Region, Northern Ethiopia	A community-based cross-sectional study was conducted in April 2013 in Tigray Region, Ethiopia.	**Survey** study.	A total of 1034 participants were enrolled in the study. A multi-stage sampling method was employed to select households.	Overall, 1000 (97%) households responded to the interview, among them 504 urban and 496 were rural. The median family size of the households was 5; just above half (52%) of the households had at least five family members. Only 7% of the surveyed households had a health professional as a family member.
2017	Teni et al. [36]	Gondar town, northwestern Ethiopia	A cross-sectional household survey was conducted from 5 April to 6 May 2015. In the study, 809 households were surveyed from four sub-cities selected through multi-stage sampling with 771 included in the final analysis.	**Survey** study.	In the study, 809 households were surveyed from four sub-cities selected through multi-stage sampling with 771 included in the final analysis.	Of the participants of the study that represented their respective households, upwards of three quarters (76.3%) and two-fifths (40.9%) were female and those in the age group of 18 to 29 years, respectively. Nearly three-fourths (73.3%) followed Orthodox Christianity, and almost all (90.3) were Amhara in their ethnic identity.
2019	Ebrahim et al. [37]	Awi zone, Amhara regional state, Ethiopia	A facility-based cross-sectional study design supplemented by a qualitative approach was conducted from 23 April to 22 May 2018.	**Survey** study.	A total of 4 health facilities were included in the study. During the 1 month of the study period, 56 types of medications were found unused at the health facilities.	Three of the heads were male and one was a female. All of them were BSc nurses with a work experience of a minimum of 4.6 and a maximum of 8 years. All the pharmacy heads were male and degree holders with a minimum experience of 4 years and maximum experience of 8 years. A total of 3 store women and 1 store man were interviewed. All the store men/women were diploma holders with a work experience of a minimum of 4 years and a maximum of 8 years.
2020	Gudeta and Assefa [39]	Jimma city, Ethiopia	A facility-based descriptive cross-sectional study was conducted among private practitioners in retail outlets of Jimma city from 20 November to 19 December 2018.	**Survey** study.	All drug shops, 35 (62.5%) and pharmacies, 21 (37.5%) in Jimma city, were visited, 3 of which were used for pre-testing. A total of 106 questionnaires were distributed to practitioners in 53 retail outlets, of which 87 returned the completed questionnaires, making a response rate of 82.1%.	The majority of the practitioners, 44 (50.6%) were within the age range of 25 to 31 years. More than half, 56 (64.4%) of them were males. Regarding their profession, the majority of them were pharmacy professionals, 73 (83.9%).
2020	Kahsay et al. [40]	Adigrat city, Ethiopia	A cross-sectional study was conducted using semistructured questionnaires, which focussed on knowledge, attitudes, and disposal practices for unused and expired medications were used to collect data from respondents.	**Survey** study.	The study was conducted among 359 respondents from the residents of Adigrat city, Ethiopia. All of the 359 returned questionnaires were valid for data entry and analysis.	All the approached 359 individuals agreed to participate in the study. Of the 359 respondents, 207 (57.7%) were males. The majority (137; 38.2%) of the respondents were 32 years old and above. Concerning their educational level, one hundred and twelve (31.2%) respondents completed secondary education, 178 (49.6%) had a college/university degree and above, and 31 (8.6%) were illiterate.
2020	Yimenu et al. [38]	Awi zone, Amhara regional state, Northwestern Ethiopia	A community-based cross-sectional study was conducted through interviews with representatives of households.	**Survey** study.	A total of 23 kebeles (the smallest an administrative unit in Ethiopia) (2 urban and 21 rural kebeles) from four woredas were selected using a multi-stage sampling technique. A total of 507 households were included in the study.	The majority of the study participants, 368 (72.6%), were female. The mean age of the study participants was 40 years, and the majority were between the ages of 30 and 65 (67.9%)

**Table A2:** Summary of the therapeutic classes, dosage forms, and limitations of the included studies.

Year of Study	Author(s)	Settings/Country	Therapeutic Category of the Unused, Wasted Medicine	Dosage Form	Study Limitation
2015	Gracia-Vásquez et al. [61]	Mexico; nine cities of Monterrey	The most commonly returned medications were of nonsteroidal anti-inflammatory followed by cardiovascular drugs. Nonsteroidal anti-inflammatory drugs: 16.11%. Cardiovascular drugs: 14.21% (Anti-hypertensive 55%). Gastrointestinal drugs 11.43%. Antibacterial drugs: 10.05%. Respiratory system drugs: 8.75%. Neurological drugs: 6.13% (anti-depressant: 34%). Dietary supplement: 5.23%. Anti-diabetic drugs: 4.34%. Miscellaneous drugs: 3.79%. Hypolipemic drugs: 3.67%. Anti-parasitic drugs: 2.48%. Hormonal drugs: 1.89%. Anti-micotic drugs: 1.84%. Steroidal anti-inflammatory drugs: 1.72%. Dermatological drugs: 1.71%. Ophthalmic drugs: 1.64%. Anti-viral drugs: 1.53%.	The majority of unused/expired medications collected (73%) was in solid dosage form (tablets, capsules, granules, powders, and lozenges). 20% were liquid pharmaceutical forms (syrups, injections, eye drops, suspensions, emulsions, and lotions). 6% were semisolid (ointments, creams, gel, paste, and suppositories). 1% were other forms, such as metered dose inhalers, sprays, patches, strips, and chewing gums.	Unable to describe respondent demographic information.
2008	Braund et al. [21]	New Zealand	The most commonly returned medications were of the nervous system drugs, followed by alimentary tract and metabolism. Nervous system drugs: 17%. Alimentary tract and metabolism system drugs: 14%. Cardiovascular system drugs: 12%. Respiratory system and allergies: 11%. Musculoskeletal system drugs: 11%. Infections—agents for systemic use: 9%. Blood and blood-forming organs: 8%. Oncology agents and immunosuppressants: 6%. Genitourinary system: 5%. Dermatologicals: 3%. Sensory organs: 2%. Hormone preparations—systemic: 2%.	Only oral dosage form reported.	Small number of returned unused medication.
2007	Braund et al. [67]	New Zealand; Otago Pharmacies	The returned medications were not classified by therapeutic group, but by generic name. The most commonly returned tablet was paracetamol (9% of all tablets returned). The most commonly returned capsule was omeprazole 20 mg (8% of capsules); additionally, omeprazole 40 mg accounted for a further 5% of all capsules.	There were 65,907 tablets returned and 7599 capsules returned. Others include injections, inhalers, eye drops, creams, gels, ointment, test strips, liquids, and suppositories.	Unable to describe respondent demographic information. Unable to report unused medicines as therapeutic group.
2009	Braund et al. [31]	New Zealand; Hutt Valley District Health Board	The predominant therapeutic group was drugs affecting the nervous system, but individually, diclofenac sodium and ibuprofen were the most returned medications, respectively. Nervous system drugs: 19%. Alimentary tract and metabolism: 13%. Cardiovascular system: 12%. Musculoskeletal system: 11%. Respiratory system and allergies, and miscellaneous: 8%. Blood and blood-forming organs: 7%, Dermatological and anti-infective: 7%. Genitourinary: 3%, Hormones: 3%.	Oral solid forms (tablets and capsules) were counted. Liquid medications were quantified by the amount left in the original container, semisolid preparations were estimated as a proportion of original container. Inhalers were recorded as either full, half-full, or empty. Anything almost empty was excluded from the analysis.	The chosen sample of the total returned unused medicine was around 20%, which maybe not representative of the whole sample.
2010	Caroline et al. [29]	New Zealand; Nelson Bays region	The most common returned (top 20) by quantities (individual unit) were (*n* = 435,397): Salazopyrin: 94,271 tablets. Paracetamol: 23,251 tablets. Lactulose: 11,324 mL. Aspirin: 10,047 tablets. Simvastatin: 7380 tablets. Diclofenac: 7014 (mixed preparation). Prednisolone: 7004 tablets. Metoprolol: 6627 tablets. Warfarin: 6590 tablets. Furosemide: 6117 tablets. Lemnis fatty cream: 6095g. Cilazapril: 5687 tablets. (Paracetamol and codeine) preparation: 5003 tablets. Ibuprofen: 4873 tablets. Codeine: 4794 tablets. Laxsol: 4267 tablets. Morphine: 4107 (mixed preparations). Emulsifying ointment: 4030 g. Quinapril: 3890 tablets.	Oral solid forms (tablets and capsules) with tablets as the most common returned dosage form. Oral liquid forms. Cream and ointment.	Unable to describe respondent demographic information.
2009	James et al. [65]	New Zealand: Taranaki region (around 37,000 households)	The predominant therapeutic group was drugs affecting the nervous system, but individually, paracetamol (acetaminophen) was the most returned medication respectively. Nervous system drugs (*n* = 658, 24.3%). Cardiovascular system (*n* = 559, 20.7%). Alimentary tract and metabolism (*n* = 529, 19.6%). Blood and blood-forming organs (*n* = 283, 10.5%). Respiratory system and allergies (*n* = 190, 7.1%).	Not studied.	Unable to describe respondent demographic information. In addition, due the different policies for collection and disposal of medicines, the majority of unused medicines were disposed into landfills and water system, which may mean that the returned amount may be underestimate of the extent of unused medicines.
2005	Langley et al. [62]	United Kingdom; East Birmingham	The predominant therapeutic group was drugs affecting cardiovascular system. Cardiovascular system drugs: 28.5%. Central nervous system drugs: 18.8%. Respiratory system drugs: 14.7%. Gastrointestinal drugs: 10.6%. Endocrine system drugs: 5.6%. Musculoskeletal and joint disease drugs: 5%. Anti-infective Drugs: 4.7%. Eye Drugs: 3.5%. Nutrition and blood drugs: 2.1%. Skin drugs: 1.8%. Obstetrics, gynaecology, and urinary tract disorders: 1.5%. Nutrition and blood and unknown: 1.2%. Malignant disease and immunosuppression: 0.9%.	Tablet or capsule, oral liquid, cream or ointment, and inhalers.	Sample size and the number of returns are small, which makes it difficult to extrapolate the result to the whole United Kingdom.
2007	Mackridge et al. [63]	United Kingdom; Eastern Birmingham Primary Care Trust (P.C.T.)	The predominant therapeutic groups were drugs affecting cardiovascular system and drugs acting on the central nervous system, respectively. The most commonly returned drugs were aspirin (102 items), co-codamol (98 items), salbutamol (96 items), furosemide (90 items), and glyceryl trinitrate (78 items). Drugs affecting cardiovascular system (1003 items, 26.6%). Drugs acting on the central nervous system (884 items, 23.5%). Drugs affecting respiratory system (358 items, 9.5%) and gastrointestinal system (358 items, 9.5%). Drugs affecting endocrine system (257 items, 6.8%). Drugs treating musculoskeletal and joint diseases (235 items, 6.2%). Anti-infective drugs (165 items, 4.4%). Drugs for skin (124 items, 3.3%). Drugs for nutrition and blood (116 items, 3.1%). Drugs for eye (65 items, 1.7%). Obstetrics, gynaecology, and urinary tract disorders (59 items, 1.6%). Drugs for ear, nose, and oropharynx (58 items, 1.5%) and others (58 items, 1.5%). Drugs for malignant disease and immunosuppression 20 items, 0.5%). Drugs for anaesthesia (5 items, 0.1%).	Tablet or capsule, oral liquid, cream or ointment, and inhalers.	The author reported that this study did not attempt to estimate the quantities of unused medicines at patient’s home; as a result, it is more likely that the unused medicines from primary care was underestimated.
2008	Bradley [64]	United Kingdom; Cumbria	The greatest value of returned of medicines was from cardiovascular and central nervous system categories (BNF), total number of returns (*n* = 4562): Cardiovascular (*n* = 1232). Central nervous system (*n* = 1149). Gastrointestinal system (*n* = 468) Endocrine (*n* = 334). Respiratory (*n* = 307). Anti-infective (*n* = 250). Musculoskeletal and joint (*n* = 228). Nutrition and blood (*n* = 141). Skin (*n* = 134). Others (*n* = 319)	Not studied.	It is an audit report with a result from Cumbria/northwest of England, which may not representative of the whole United Kingdom and may underestimate the extent of unused medicines.
2010	Trueman et al. [6]	United Kingdom	Coding was based on guidance provided by the Royal Pharmaceutical Society of Great Britain/BNF. The most common retuned medication was for the cardiovascular and central nervous system. Cardiovascular system drugs (1950 items, 22.6%). Central nervous system drugs (1907 items, 22.11%). Gastrointestinal system drugs (828 items, 9.6%). Respiratory system drugs (528 items, 6.12%). Endocrine system drugs (518 items, 6.01%). Endocrine system drugs (518 items, 6.01%). Anti-infective drugs (444 items, 5.15%). Musculoskeletal, joint disease drugs (364 items, 4.22%). Nutrition and blood drugs (249 items, 2.89%). Skin drugs (192 items, 2.23%). Eye drugs (129 items, 1.5%). Ear, nose, oropharynx drugs (68 items, 0.79%). Malignant disease and immunosuppression drugs (53 items, 0.61%). Wound management drugs (34 items, 0.39%). Borderline substances (25 items, 0.29%). Drugs for Anaesthesia (9 items, 0.10%).	Not studied.	Unable to describe respondent demographic information.
2008	Coma et al. [56]	Spain; Barcelona	The predominant therapeutic groups were drugs affecting the alimentary tract and metabolism, nervous system, and cardiovascular system, respectively. All drugs were categorised according to Anatomical Therapeutic Chemical (A.T.C.) system/code of the World Health Organisation (WHO). Alimentary tract and metabolism drugs (215 items, 18.3%). Nervous system drugs (214 items, 18.2%). Cardiovascular drugs (137 items, 11.6%). Respiratory system drugs (103 items, 8.8%). Musculoskeletal system drugs (88 items, 7.5%). Dermatological drugs (85 items, 7.2%). Anti-infective drugs (77 items, 6.5%). Missing drugs (could not be coded according to the A.T.C. system), (66 items, 5.6%). Sensory organs drugs (63 items, 5.4%). Drugs affecting genitourinary system and sex hormones (50 items, 4.3%). Drugs affecting blood and blood-forming organs (32 items, 2.7%). Antineoplastic and immune-modulating drugs (22 items 1.9%). Systemic hormonal preparations excluding sex hormones and insulins, (17 items, 1.4%). Various drugs (5 items, 0.4%). Anti-parasitic products, insecticides, and repellents (2 items, 0.2%).	Not studied.	Unable to describe the respondent demographic information clearly.
2015	Law et al. [46]	U.S.A.; Southern California	Approximately 2 of 3 prescription medications were reported unused. In Phase I, pain medications (23.3%) and antibiotics (18%) were most commonly reported as unused. In Phase II, 17% of medications for chronic conditions (hypertension, diabetes, cholesterol, heart disease) and 8.3% for mental health problems (antidepressants/antipsychotic/anti-convulsant) were commonly reported as unused. 7% painkillers and 4% electrolytes and dietary supplements.	Tablets, pills, capsules, and liquid preparations.	Use of a web-based survey may limit the accessibility of this study to people without computer and Internet access at home, which may to some extent underestimate the extent of unused medicines. Unable to describe respondent demographic information.
2004	Garey et al. [25]	U.S.A.; Houston, Texas	The predominant therapeutic group was nonsteroidal anti-inflammatory drugs/pain. Nonsteroidal anti-inflammatory drugs/pain 25%. Drugs for cough/cold/allergy 15%. Anti-infective drugs 11%. Cardiovascular drugs 10%. Respiratory drugs 9%. Neurological drugs 8%. Dermatological 7% and gastrointestinal 7%.	Oral medications (capsules or tablets) were most commonly returned (64%), followed by liquid (12%), creams (11%), inhalers (7%), or miscellaneous (6%; e.g., eye glasses, hearing aid batteries, medical equipment). Approximately 17,000 oral pills were collected during the study period.	Unable to describe respondent demographic information.
2016	Maeng et al. [34]	U.S.A.; Regional health plan in Central Pennsylvania	The predominant therapeutic group was pain medication (15%), hypertension (14%), antibiotics (11%), and psychiatric disorders (9%).	Not studied.	Unable to describe respondent demographic information.
2014	Vogler et al. [66]	Austria; Vienna	The predominant therapeutic group was cardiovascular drugs. Cardiovascular drugs (36 packs, 23.7%). Musculoskeletal system drugs (17 packs, 11.2%). Nervous system drugs (16 packs, 10.5%) Alimentary tract and metabolism 15 packs, 9.9%). Anti-infective drugs for systemic use (5 packs, 3.3%). Drugs for blood and blood-forming organs (4 packs, 2.6%). Genitourinary system drugs and sex hormone (2 packs, 1.3%) and Dermatological drugs (2 packs, 1.3%). Other A.T.C. code or not attributable (45 packs, 29.6%).	Oral medications were the most commonly founded 86.8% (usually solid oral), followed by dermal 6.7%, parental 4%, nasal 0.7%, pulmonary 0.7%, eye 0.7%, and dental 0.7%.	Unable to describe respondent demographic information.
2013	Chien et al. [71]	Taiwan; Shuang-Ho university teaching hospital	Among the discarded medications, gastrointestinal drugs were at the top of the list of all discarded medications. The analysis of discarded and unused drugs revealed that Strocain (oxethazaine, polymigel) was on top of the list, followed by Glucobay (acarbose), Mopride (mosapride), and Loditon (metformin). Gastrointestinal drugs: 25.93%. Cardiovascular drugs: 22.49%. Anti-inflammatory drugs: 12.15%. Anti-diabetic drugs: 9.49%. Cold medicines: 6.83%. Psychiatric drugs: 5.44%. Respiratory drugs: 2.16%. Rheumatological drugs: 1.52%. Antimicrobial drugs: 1.42%. Others: 9.19%. Health foods: 3.38%.	Tablets, bottles, and tubes.	Unable to describe respondent demographic information.
2013	Abushanab et al. [48]	Jordan; Amman	Alimentary tract and metabolism drugs were the most commonly found in household (both used and unused). Stored drug products were classified by A.T.C. code of WHO. Alimentary tract and metabolism: 519 (20.7%). Nervous system: 370 (17.3%). Musculoskeletal system: 313 (12.9%). Respiratory system: 291 (12%). Cardiovascular system: 256 (10.9%). Anti-infective for systemic use: 252 (10.6%). Dermatological: 149 (5.4%). Blood and blood-forming organs: 109 (4.6%). Genitourinary system and sex hormones: 31 (1.1%). Systemic hormonal preparations, excl. sex hormones and insulin: 18 (1.1%). Anti-parasitic products, insecticides and repellents: 13 (0.7%). Anti-neoplastic and immune-modulating agents 8 (0.3%), sensory organs 63 (2.5%).	Not studied.	Studied the medication stored at home the estimated the unused wasted medicine as the sum of drug products that had expired, had no clear expiration date, or which had never been used since dispensing. So not directly investigate the unused wasted medicine.
2012	Al-Azzam et al. [47]	Jordan; North of Jordan particularly Irbid	Central nervous system drugs were found to be the most common, followed by anti-infective agents. The most common individual medications found were amoxicillin, paracetamol, metronidazole, antihistamines, hypoglycaemic medications, and adult cold medications. Central nervous system drugs (713 items, 25.2%). Anti-infective agents (493 items, 17.4%). Musculoskeletal agents (381 items, 13.4%) Respiratory system agents (348 items, 12.3%) Gastrointestinal agents (301 items, 10.6%) Cardiovascular agents (216 items, 7.6%) Endocrine system agents (200 items, 7.0%) Nutrition agents (127 items, 4.5%). Eye, ear, nose and skin agents (56 items, 2.0%).	Tablets (1794 items, 63.3%) Capsules (332 items, 11.7%) Syrups (250 items, 8.8%) Suspensions (201, 7.1%) Suppositories (117 items, 4.1%) Creams/ointments/gels (43 items, 1.5%) All forms of injections (53 items, 1.9%) Drops/nasal or oral puff (45 items, 1.6%).	A sample was selected from northern Jordan, which may not representative of the whole of Jordan.
2002	Abou-Auda [27]	5 regions in Saudi Arabia and other Gulf countries (Kuwait, U.A.E., Qatar, and Oman)	Medications were also categorised according to their pharmacologic or therapeutic class using the classification of drugs adopted in the Saudi National Formulary (SNF). Respiratory system drugs Saudi Arabia: 2095 (16.8%), other gulf countries: 94 (15.3%). Central nervous system drugs Saudi Arabia: 2050 (16.4%), other gulf countries: 84 (13.6%). Antibiotics Saudi Arabia: 1779 (14.3%), other gulf countries: 111 (18.0%). Gastrointestinal drugs Saudi Arabia: 1382 (11.1%), other gulf countries: 60 (9.7%). Miscellaneous Saudi Arabia: 847 (6.8%), other gulf countries: 57 (9.3%). Nutrition and blood drugs Saudi Arabia: 823 (6.6%), other gulf countries: 24 (3.9%). Musculoskeletal/joints drugs Saudi Arabia: 790 (6.3%), other gulf countries: 52 (8.4%). Skin drugs Saudi Arabia: 735 (5.9%), other gulf countries: 33 (5.4%). Ear, nose, and throat drugs Saudi Arabia: 553 (4.4%), other gulf countries: 26 (4.2%). Cardiovascular drugs Saudi Arabia: 465 (3.7%), other gulf countries: 60 (9.7%). Eye drugs Saudi Arabia: 398 (3.2%), other gulf countries: 25 (4.1%). Endocrine drugs Saudi Arabia: 375 (3.0%), other gulf countries: 16 (2.6%). Obstetric/gynaecologic and/or urinary drugs Saudi Arabia: 140 (1.1%), other gulf countries: 12 (1.9%). Cytotoxic drugs Saudi Arabia: 31 (0.2%), other gulf countries: 0 (0.0%). Total drugs Saudi Arabia: 12,463 (100%), other gulf countries: 616 (100%). The mean medication wastage was estimated to be 25.8% Saudi Arabia and 41.3% other gulf countries.	Not studied.	Unable to describe respondent demographic information.
2011	Kheir et al. [51]	Qatar	The majority of the drugs stored (*n* = 58; 21%) in the participating homes were analgesics. Nonsteroidal anti-inflammatory drugs were the second most commonly stored drugs, representing 16% of all the drugs.	Not reported.	There was potential for selection and social desirability bias as a result of the strategy of using the telephone to conduct an interview. In addition, interviews were conducted during working hours, which could run the risk of excluding highly educated young subjects. Due to the small sample size, the results of this exploratory study should be considered with caution.
2007	Al-Siyabi et al. [60]	Oman; Sultan Qaboos University Hospital (SQUH)	Cardiovascular drugs were the most common pharmacological group of returned drugs. The drugs were classified according to the classification index of the British National Formulary. Cardiovascular drugs: 24%. Central nervous system drugs: 14%. Anti-infective drugs: 13%. Endocrine drugs: 10%. Nutrition: 9%. Gastrointestinal drugs: 8%, and Musculoskeletal system drugs: 8%. Respiratory system drugs: 5%. Immunosuppressant drugs: 3%. Eye/Ear drugs: 2%.	Not studied.	Unable to describe respondent demographic information. As it included only medicines with SQUH labels, others were missed, and this may underestimate the extent of unused medicines.
2004	Wongpoowarak et al. [20]	Thailand; Songkhla	Musculoskeletal system drugs were the most common pharmacological group of returned drugs. The medications were pharmacologically classified using MIMS Thailand, which is a standard reference source. Musculoskeletal system drugs (229 items, 23.3%). Anti-infective drugs (189 items, 19.2%). Respiratory system drugs (166 items, 16.9%). Gastrointestinal system drugs (129 items, 13.1%). Allergy and immune system drugs (91 items, 9.2%). Vitamins and minerals (68 items, 6.9%). Others (54 items, 5.5%). Central nervous system (37 items, 3.8%). Cardiovascular (21 items, 2.1%).	Oral dosage forms compromised 95.6% (951 items). Oral tablets or capsules (636 items, 63.9%). Oral liquids (311 items, 31.3%). Eye drops (23 items, 2.3%). Topical liquids (14 items, 1.4%). Creams (5 items, 0.5%). Oral powders (4 items, 0.4%). Inhalers (2 items, 0.2%).	This study was a snapshot study, as the studied population was one of 14 provinces in southern Thailand.
2013	Sooksriwong et al. [50]	Thailand; 4 regions of Thailand: Bangkok, Chiang Mai, Khon Kaen, Mahasarakham and Songkla	A total of 2208 drug items found in household surveys were classified into 5 groups of the mostly found drugs. These were 343 non-opioid analgesics and antipyretic drugs, 188 antacids, anti-reflux agents and anti-ulcer, 180 nonsteroidal anti-inflammatory drugs (NSAIDs), 127 antihistamine and anti-allergic and 119 anti-diabetic drugs. Top 5 of the most found rarely or unused drugs, classified as leftover medicines, were NSAIDs (49 items), penicillin (38 items), G.I.T. regulators, and antiflatulents (36 items). Of the total of 2208 drug items found in household, 82 items (3.7%) and 45 items (2.0%) of drugs were already expired and deteriorated, respectively.	Not studied.	Unable to describe respondent demographic information.
2011	El-Hamamsy A [26]	Egypt; Cairo	The returned medications were classified according to the British National Formulary (BNF). Antibiotics were the most common pharmacological group of returned medications. Antibiotics (109 items, 20.15%). Gastrointestinal system drugs (88 items, 16.27%). Cardiovascular system drugs (58 items, 10.72%). Respiratory system drugs (44 items, 8.13%). Nervous system drugs (39 items, 7.20%). Analgesics and anti-inflammatory (38 items, 7.02%). Dermatological drugs (35 items, 6.47%). Blood and blood-forming organs (29 items, 5.36%). Systemic hormonal preparations, sex hormones, and insulin’s (27 items, 4.99%). Anti-parasitic products, insecticides, and repellents (25 items, 4.62%). Genitourinary system (20 items, 3.69%). Antineoplastic and immune-modulating agents (3 items, 0.55%). Various others (26 items, 4.80%).	Not studied.	Unable to describe respondent demographic information.
2012	Ibrahim et al. [49]	Egypt; Alexandria	Cardiovascular system drugs were the most common pharmacological group of returned medications. The returned medications were classified according to the British National Formulary (BNF). Cardiovascular system (127 items, 19.4%). Anti-infective (126 items, 19.2%). Gastrointestinal system (66 items, 10.9%). Nutrition and blood (69 items, 10.6%). Nonsteroidal anti-inflammatory (64 items, 9.8%). Nervous system (61 items, 9.3%). Respiratory system (58 items, 8.9%). Endocrine System (49 items, 7.5%). Skin care (19 items, 2.9%). Ear, nose, throat (7 items, 1.1%) and genitourinary system (7 items, 1.1%). Musculoskeletal system (2 items, 0.3%).	Not studied.	This study did not estimate the quantities of unused medicines in patient’s home. As result, it is likely that it may underestimate the extent of unused medicines in the community.
2010	Guirguis et al. [32]	Australia; St Vincent’s Hospital, Melbourne	Cardiovascular system drugs were the most common pharmacological group of returned medications. The smallest group was that of topicals, e.g., creams and ointments. Cardiovascular system drugs (78 items, 26.6%). Analgesics/anti-inflammatories (62 items, 21.2%). Neuropsychiatry drugs (8.5%). Respiratory system drugs (8%). Eye/Ear/Nose drugs (7.5%). Gastrointestinal drugs (7%), and Antimicrobials (7%). Herbals and vitamins (12 items, 4.1%). Diabetes drugs (3%). Topicals, e.g., creams and ointments (8 items, 2.7%). Miscellaneous (4.5%).	They report that they collect topicals cream, ointment along with other dosage forms (that was not defined).	Sample size and the number of returns are small, which make it difficult to extrapolate the result to the whole of Australia.
2014	Kagashe et al. [57]	Tanzania; tertiary hospital in Dar es Salaam city	Medicines wasted in this study were categorised into three major groups, anti-infective, cardiovascular medications, and others. Anti-infective drugs: 18.9%. Cardiovascular drugs: 8.9%. Other drugs: 23.7%.	Oral solids drugs were the most common wasted dosage form 40.6% followed by injections 9.2%, with very few topicals preparations.	Since only hospital-prescribed medicines was included, others may be missed, which may underestimate the extent of unused medicines.
2007	Abahussain et al. [33]	Kuwait; Kuwait city	No medicines were collected from the 200 households participating in the municipal collection program The second intervention yielded 123 medicines from 14 homes; the most common class of unwanted medicines were drugs for respiratory system. Unwanted medications were classified according to the ATC WHO classification. A third of all unwanted medicines were for the respiratory system (38% of these were cough and cold preparations, 25% nasal preparations). 12% of the medicines were for the musculoskeletal system (53% oral NSAIDs) or were dermatologicals (33% topical antibiotics).	There were 141 items (including duplicates). 508 tablets/capsules, 25 oral liquids, 20 tubes, 21 dropper bottles, and various other dosage forms.	Sample size and the number of returns are small, which make it difficult to extrapolate the result to the whole of Kuwait. Unable to describe respondent demographic information.
2013	Aditya [43]	India; dental hospital in North India	Qualitative analysis of expired medications at home revealed antipyretics (54%), analgesics (64%), followed by antihistamines (35%) to be hoarded in home pharmacies/medicine chests. Other drugs were antibiotics (26%), antacids (23%), topical drugs (39%) and supplements (vitamins) (41%). Excessive buying of over-the counter (O.T.C.) drugs (53%); self-discontinuation (17%), and expiration of drugs (24%) resulted in possession of unused/leftover medications at home.	Not studied.	Small sample size from a specific region in India, which make it difficult to generalise and extrapolate the results to the whole of India.
2011	Gupta et al. [42]	India; Greater Noida City	Most of the expired drugs are in the category of analgesics and NSAIDs (23.93%) followed by nutritional supplements (22.56%), antibiotics (14.94%), expectorants and mucolytics (6.77%), bronchodilators (5.31%), and antacids (6.53%).	Oral tablets were the most common; other dosage forms include syrups, capsules, suspensions, powders, eye drops, gels, churna, cream, and ear wax softener.	Defined medicine wastes as only expired medicines, which may underestimate the extent of unused wasted medicines.
2014	Mirza and Ganguly [44]	Anand district of Gujarat, India	Among the prescribed medicines, the majority of medicines were from cardiovascular disease (19.88%) and from without prescription medicines, nonsteroidal anti-inflammatory drugs (NSAIDs) were the major group available at houses (35.13%).	Not reported.	Since the interviewers were fully aware of the purpose of the project, some information regarding medicines was not shared, which might have led to a skewed result.
2009	Ali et al. [24]	Malaysia; Universiti Sains	The total number of medicines found unused was 1724 drug products with vitamins and minerals as the most common class of unused drugs. Vitamins and minerals: 427 (24.8%). Gastrointestinal drugs: 298 (17.3%). Analgesic and antipyretics: 293 (17.0%). Antibiotics: 174 (10.0%). Ear, nose, and throat drugs: 159 (9.2%). Respiratory drugs: 106 (6.3%). Dermatological products: 97 (5.6%). Anti-rheumatic and anti-inflammatory: 69 (4.0%). Others (C.N.S. drugs, endocrine and metabolic drugs, cardiovascular drugs, genitourinary drugs, and others): 101 (5.8%).	68.5% (*n* = 1181) of the medications were in the form of tablets and pills while capsules constituted 14.6% (*n* = 252) of the overall amount. 5% (*n* = 87) syrups and suspensions while 4.9% (*n* = 84) were creams and ointments. Less than 1.0% (*n* = 5) consisted of inhalers, with 0.2% (*n* = 4) suppositories of the overall total.	Sampling of only female students made it impossible to generalise the results to the whole student population in the campus.
2020	Hassali and Shakeel [45]	Selangor, Malaysia	The major classes of medications that were purchased included antibiotics (207; 48.5%) followed by painkillers/nonsteroidal anti-inflammatory drugs (NSAIDs) (101; 23.7%). In addition, anti-hypertensive 51 (11.9%), anti-diabetic 20 (4.6%), OTC antihistamines 34 (7.9%), and multi-vitamins and other supplements 13 (3.0%).	Not studied.	The sample size of the study was small to depict a clear picture of the entire Selangor population; hence, the findings of the current study are not generalisable to all of Malaysia.
2014	Aboagye et al. [59]	Ghana	Leftover medicines: Paracetamol tablets 27 Amoxicillin capsules 12 Aspirin tablets 4 Metronidazole tablets 5 F-PAC (Paracetamol/Aspirin/Caffeine) 3 Vitamin B complex tablets 7 Multi-vitamins tablets 7 Diclofenac tablets 3 Magnesium trisilicate tablets 3 Ibuprofen tablets 5 Others/Unidentified 45 Do not remember 1.	Not studied.	Sample size and the number of returns are small which make it difficult to extrapolate the result to the whole of Ghana. Leftover medicines were described as individual medicine, not as a group.
2019	Huang et al. [52]	Six provinces in North, Central, and Southern regions of China	Cold medication (86.1%) was the most common category of medicines kept in households. Specifically, the following were the major classes of medicines found in the households: gastrointestinal medicines (27.0%), pain medications (22.9%), vitamins (20.6%), antibiotics (19.0%), external painkillers (16.5%), and external anti-inflammatory antidotes (15.4%).	Not studied.	Not reported.
2019	Vella and West [58]	Maltese village, Malta	The most common class of disposed medications was that pertaining to the alimentary tract (24.6%), closely followed by medicines belonging to the respiratory group (23.8%). 10.5% of the unused disposed medications were from the musculoskeletal group, which includes medications such as nonsteroidal anti-inflammatory drugs (NSAIDs), and supplements, such as glucosamine. The medications with the lowest return rate were anti-neoplastic and immunomodulating agents (0.7%), followed by anti-parasitic medications (0.2%).	Solid dosage forms were counted manually, liquid dosage forms were measured using a calibrated measuring cylinder, dermatological preparations were measured using kitchen weighing scales, and inhalers that had a counter were recorded as per value available on the counter. Unused inhalers without a counter, eye drops, ear drops, nasal drops, and nasal and oral sprays were not quantified as effective entries, as their quantities could not be safely determined.	This study excluded some dosage forms whilst quantifying and costing waste, such as eye drops, inhalers, and nasal sprays. Therefore, the actual cost of waste presented in this study is an underestimate.
2020	Insani et al. [54]	Bandung, Indonesia	NSAIDs were the most common medicines left unused (*n* = 372) followed by vitamins and nutritional supplements (*n* = 215) and antibiotics (*n* = 171).	Not reported.	This study was conducted in one region in Bandung (small sample size); thus, its generalisation for the Indonesian population is limited. In addition, the predictors associated with disposal practice were not identified.
2010	Jassim [53]	Basrah, Iraq	Overall, 4279 items of drugs were analysed. Antibiotics were the leading household stored drugs (26.43%), followed by antipyretic/analgesics (19.58%), and NSAIDs (nonsteroidal anti-inflammatory drugs) (11.45%). These drugs constituted (57%) of the total drugs stored.	Not reported.	This study was conducted in 300 households in Basrah, southern Iraq (i.e., one region in Iraq). Small sample size.
2012	Auta et al. [55]	Nigeria	Common classes of medicines reported as leftover medicines were analgesics (36.4%), antibiotics (33.1%), and antihistamines (11.9%).	Respondents reported having about 318 medicines items (representing 2.56 items per student’s room) in all, with the tablets (62.3%) being the most common dosage form. Followed by capsules (16.4%), lotions/creams (11.6%), and syrups/suspensions (6.3%).	This study was based on the self-reported presence of medicines in respondents’ residence. Therefore, it is possible that the medicines were under-reported or some names of unidentified medicines were wrongly reported. In addition, the sample size was small.
2015	Wondimu et al. [41]	Tigray Region, Northern Ethiopia	The most common classes of drugs found in the households were analgesics (29%) and antibiotics (25%). Generally, more than half (62%) of the medications were used for ongoing treatment.	Most (70%) of the medicines were available in the form of tablets, followed by capsules (13.2%), oral liquid (9.9%), semisolids (2.8%), injections (1.8%), and other dosage forms (2.2%).	One of the study limitations was the cross-sectional design employed, which might be affected by temporal relationship establishment with some variables and could not provide much more substantial evidence of causality, unlike a longitudinal design.
2017	Teni et al. [36]	Gondar town, northwestern Ethiopia	Anti-infectives for systemic use (23.9%), medicines for alimentary tract and metabolism (19.2%) and those for the cardiovascular system (17.7%) ranked top.	Of the total 553 medicines stored, more than three quarters (80.8%) were of solid dosage forms. Liquid dosage forms were (16.6%) and semisolids were (2.5%).	The study did not include the rural parts of Gondar Town. The small sample size makes the findings not representative of the pattern of household medicine storage practice in those areas.
2019	Ebrahim et al. [37]	Awi zone, Amhara regional state, Ethiopia	Anti-infective medications were found to be the most frequently unused medications 63 (36.4%) followed by antipain medications 37 (21.4%) and cardiovascular medications 19 (11%).	Not reported.	Health centres and private health facilities were not included in the study, and thus, the results may have been slightly different if those facilities were included.
2020	Gudeta and Assefa [39]	Jimma city, Ethiopia	Antibiotics, 31 (35.6%), and anti-hypertensive, 21 (24.1%) constituted the highest proportion of the waste.	Not reported.	The sample size was small. In addition, the current study was conducted among private practitioners. Thus, prospective researchers may consider both private and public professionals for their comparative study.
2020	Kahsay et al. [40]	Adigrat city, Ethiopia	The common types of medicines kept in households were analgesics (41.5%) and antibiotics (36.7%). In addition, antipain and antibiotic (4.8%), anti-diabetic (5.3%), and anti-hypertensive (8%) medicines were other types of unused medications found in homes.	Not reported.	The small sample size and the cross-sectional nature of the study design prevent us from drawing causal inferences about the relationship between the chosen covariates and outcome variables over a period.
2020	Yimenu et al. [38]	Awi zone, Amhara regional state, northwestern Ethiopia	Anti-infective medicines were found to be the most common unused medicines, 53 (58.9%), followed by antipain medicines, 16 (17.8%).	Not reported.	The small sample size and not including the health centres and private health facilities were limitations to this study. Thus, the results may be slightly different if those facilities were included.
